# Teaching anatomy using an active and engaging learning strategy

**DOI:** 10.1186/s12909-019-1590-2

**Published:** 2019-05-16

**Authors:** Keerti Singh, Ambadasu Bharatha, Bidyadhar Sa, Oswald Peter Adams, Md. Anwarul Azim Majumder

**Affiliations:** 1 Faculty of Medical Sciences, The University of the West Indies, Cave Hill, Barbados; 2 Faculty of Medical Sciences, The University of the West Indies, St. Augustine, Trinidad and Tobago

**Keywords:** Anatomy, Student-centred learning strategies, Active and engaging learning, Medical education, Barbados

## Abstract

**Background:**

Various evidence-based and student-centered strategies such as team-based learning (TBL), case-based learning (CBL), and flipped classroom have been recently applied to anatomy education and have shown to improve student engagement and interaction. These strategies shift the focus of teaching from knowledge transmission to knowledge construction by students and encourage the use of tasks. This study discusses the use of an active and engaging learning strategy to teach the musculoskeletal system to Year 1 MBBS students (Faculty of Medical Sciences, The University of the West Indies, Cave Hill, Barbados) and examines the correlation between assessment modalities and student performance.

**Methods:**

The “Active and Engaging Learning Strategy” was used to assess student learning in the form of oral presentations. Students had presentations on muscle attachments, muscle actions, blood and nerve supply, and applied anatomy of the limb musculature. Questions on the limbs (Locomotor System) were included in pre and post-presentation spotters, in-course assessments, and final examinations. Percentages, paired t-test, independent sample t-test, and zero-order correlations were performed to confirm the results for the different objectives of the study.

**Results:**

The main modes of presentation chosen were poems (37.1%), followed by stories (21.2%), songs (11.4%), and skits (10.6%). The majority of students (84%) found the strategies beneficial and recommended such sessions for future cohorts (92%). Students achieved significantly better scores in post-presentation spotters (*p* < 0.01) and the marks of in-course and final examinations also showed significant improvement (*p* < 0.01).

**Conclusion:**

Our study highlighted that the active and engaging learning strategy can be used as an effective learning tool in anatomy. Students were proactive in preparing the muscle presentations by utilizing their own creativity, curiosity, and intelligence. Further studies should be conducted using randomized controlled trials to assess the effectiveness of various learning strategies which could open a new door to medical education.

**Electronic supplementary material:**

The online version of this article (10.1186/s12909-019-1590-2) contains supplementary material, which is available to authorized users.

## Background

Anatomy is usually considered to be the ‘foundation of medical sciences’ [1–3], but it is also perceived to be an onerous and challenging subject in medical education [4]. Medical students need to acquire core anatomical knowledge to build a strong foundation for future clinical encounters and professional practice [2, 5]. Traditionally, anatomy was considered to be a dull, labour-intensive subject, and was taught using surface learning approaches and rote memorization [6, 7]. However, the teaching of anatomy is undergoing an evolutionary change with the adoption of modern philosophies, approaches, and effective teaching and learning strategies. An important point of concern is, anatomy teaching in the medical and other health professional education programmes is on the decline and ‘has fallen below a safe level’ in recent years [2, 8]. Furthermore, anatomists face the recent pressures of the changing modes of medical education and assessment, with fewer contact hours and limited resources to teach an extremely diverse group of students with different sets of prior scientific literary levels, cultural backgrounds, and experiences [9, 10]. Thus, it is important for anatomists to explore innovative and stimulating, engaging, creative, purposeful, multimodal means to encourage proactive and deep learning, and to develop long-term memory in students in order to improve student engagement and learning outcomes that align with their professional goals [3, 11, 12]. Though anatomy classrooms use a wide range of technology in the form of e-textbooks, models, CD-ROMs and simulations, engaging modern and alternative approaches in medical education has become an essential element today [13, 14]. New strategies are emerging in anatomy teaching that incorporate technology, making learning interactive, student-centered, and more appealing to the general student body [13–15].

Learning anatomy with cadaveric dissection has become almost non-existent in most medical schools owing to the shortage in the number of cadavers compared to the growing student numbers [16]. In recent years, a paradigm shift from teacher-centered to a learner-centered approach has been observed in teaching and learning of anatomy using innovative and interactive instructional strategies and techniques [2, 5, 13–15, 17]. Team-based learning, case-based learning (CBL), flipped lectures, peer teaching, computer-assisted learning, videos, simulations, and virtual interactive 3D images are a few examples of such approaches. These approaches make anatomy teaching interesting, interactive, and engaging, and help students with deep learning, effective retention, and application of the knowledge in the clinical context [2, 5]. Moreover, these strategies also mitigate challenges associated with teaching anatomy such as a shortage of anatomists [18], lack of cadaveric materials [13, 16], and the excessive content in the anatomy curriculum [1, 8, 9, 19–21]. As a result, the perception of anatomy learning has gradually changed from a dry, boring subject to an engaging and a fascinating subject as student-centered approaches are adopted to teach and assess the knowledge of anatomical structures [4].

Students must receive adequate and appropriate training in musculoskeletal anatomy because musculoskeletal complaints are found to be the most common presenting illnesses in primary care settings in many developed and developing countries [22]. Moreover, studies have demonstrated significant deficits in musculoskeletal education, resulting in a low competency in performing musculoskeletal physical examinations, and a lack of confidence in interpreting the findings [23, 24]. In the United States Medical Licensing Examination (USMLE) Step 2 Clinical Skills Examination, performance of the musculoskeletal physical examination is significantly poorer in comparison to physical examination performance in other domains [25]. To reduce these shortcomings, innovative approaches must be used in undergraduate medical education to improve the quality of teaching and learning of the musculoskeletal system [26].

In order to improve teaching of the musculoskeletal system in anatomy to Year 1 MBBS students of the Faculty of Medical Sciences (FMS), The University of the West Indies (UWI), Cave Hill Campus, Barbados, we designed an ‘active and engaging learning strategy’ based on the definitions of active learning by Bonwell & Eison [27] and Felder and Brent [28]. Bonwell & Eison [27] defined active learning as “anything that involves students in doing things and thinking about the things they are doing”, and Felder and Brent [28] mentioned active learning as “anything course-related that all students in a class session are called upon to do other than simply watching, listening, and taking notes”. The “Active and engaging learning strategy” shifts the focus of teaching away from knowledge transmission to knowledge construction by students and encourages the use of tasks, interactive presentations, assignments, and creative activities. In this study, the aim was two-fold: (i) to report students’ experience with the student-centered ‘active and engaging learning strategy’ introduced to teach the musculoskeletal system, and (ii) to determine if there was a relationship between assessment modalities and student performance.

## Methods

### Ethical approval

Ethical approval for the study was obtained from the Institutional Review Board (IRB of The UWI Cave Hill Campus and the Ministry of Health, Barbados (IRB No: 170203-B).

### Study participants and settings

The study was conducted in the 2017/2018 academic year. Sixty-six first-year students registered in the Locomotor System course were invited to participate in the study. The Locomotor System course is a 13-week course taught in semester one, Year 1 of the MBBS programme. It is a part of an integrated series of courses in the pre-clinical phase (3 years) of the MBBS programme (5 years). This course is usually delivered utilizing multimodal approaches such as lectures, seminars, tutorials, demonstrations, laboratory practicals, case/problem-based learning, simulations, use of multimedia, and the University’s E-learning Course Management System (Moodle). Before the introduction of the active and engaging learning strategy, anatomy teaching was mostly done using didactic lectures by PowerPoint delivery, prosected specimens, CD-ROMs, and CBL.

Grading of the Locomotor System course consisted of: (1) Continuous assessments (40%) comprising of spotters (10%), presentations using the active and engaging learning strategy (10%), and a mid-term exam (20%), and (2) a final exam (60%) using multiple choice questions (MCQ) and short answer questions (SAQs).

### Study design

- *Active and engaging learning and assessment strategies*

We developed an ‘active and engaging learning strategy’ based on Bonwell & Eison’s [27] and Felder and Brent’s [28] active learning concepts and introduced it in the Locomotor System course to teach the musculoskeletal system in Semester 1. Each student was invited to create upper (UL) and lower limbs (LL) muscle presentations using a poem, story, song, skit, video, poster, PowerPoint presentation etc., which would be engaging and interesting to their peers. Student performance in the pre-presentation spotter assessment was compared with the post-presentation spotter assessment, mid-term, and final examinations to assess the overall effectiveness of the active and engaging learning strategy.

b. *Upper limb muscles presentation*
  i. *Pre-presentation spotter (week 7)*

Students were given an unscheduled UL anatomy spotter exam in week 7 to assess their baseline knowledge of the muscles. There were 20 SAQs in the spotter. These pre-test marks did not contribute to the final grades for the course. At the end of the pre-test, students were allocated muscles to be the subject of their presentations in week 8.

Students were randomly divided into two batches Group A and Group B (33 students in each) for muscle presentations in the anatomy lab. Fifty-one muscles were identified for presentation. Each student was allotted one or a maximum of two muscles. The time limit for each presentation was 5 minutes. Students were instructed to prepare a presentation using an active and engaging learning strategy of their choice (poem, song, game, skit, role play, video, drawing, monologue, etc.) highlighting the attachments, actions, blood, nerve supply, and clinical anatomy of the muscles allocated to them. They were given a short briefing to introduce the options for the various modes of muscle presentations and regarding the five main scoring criteria included in the scoring rubric. They were also advised to discuss the various presentation modes with their seniors (Year 2 MBBS students) who underwent a similar exercise in the previous year but were not formally assessed.

ii. *Upper limb muscles presentation session (week 8)*

Students had their upper limb muscle presentations using a pre-designed scoring rubric (Additional file 1) explaining the mark distribution for creativity, relevance, accuracy, completeness, and confidence of the student (each component was worth 2 marks for a maximum score of 10). These marks were worth 5% of the final course grade.

iii. *Upper limb post-presentation spotter (week 9)*

The post-presentation spotters for both Group A and Group B were held in week 9 (1 week after the UL presentations) on the same day for both groups. It contributed 5% of the final course mark for the students. The spotter included 20 SAQs related to the muscles presented in week 8.

c. *Mid-term exam (week 9)*

A mid-term examination consisting of 40 MCQ questions and contributing 20% of the final course mark was also held in week 9.

d. *Lower limb muscles presentation session (week 11)*

There were 49 muscles identified for presentation. The same procedures were used in the allocation and presentation of the muscles as in Week 9. This presentation was also worth 5% of the final course grade.

e. *Lower limb post-presentation spotter (week 12)*

The LL spotters followed the same guidelines as UL. The only difference was, there was no pre-presentation spotter. There were 25 SAQs in the spotter, these were related to the muscles presented in week 11. The post-presentation spotter contributed 5% of the final course marks for the students.

f. *Final examination:*

Final examinations were held in week 14. The exam paper consisted of 50 MCQs and 4 SAQs testing knowledge of UL and LL muscles. The weighting of the exam was 60% of the final mark of the course.

### Feedback from the students (students’ experience)

An online questionnaire using Survey Monkey was used to collect data on the students’ experience with the active and engaging learning strategy. The questionnaire consisted of six questions related to the muscle presentations; three were closed-ended and three open-ended questions. The questionnaire was developed by the authors and reviewed by experts from the anatomy and medical education departments.

### Statistical analyses

All analyses were performed using SPSS 24 Statistical software. Percentages were calculated to ascertain the students’ experience with the active and engaging learning strategy used for the muscle presentations. A paired t-test was used to establish whether there was a significant difference between pre- and post-presentation performance, and an independent sample t-test was calculated, to discover whether there was a significant difference between Group A and Group B’s performance in different components of assessment. Zero-order correlations were performed to determine whether any correlations existed between the UL post-presentation spotter (week 9), mid-term exam (week 9), LL post-presentation spotter (week 11), and final examination (week 14) scores.

## Results

### Modes of presentations used for active and engaging learning strategy

Sixty-six Year 1 students (response rate 100%) attended the muscle presentation sessions using active and engaging learning strategies, spotters, and examinations. Attendance was mandatory in these sessions. Poems (37.1%) were most frequently used in making the UL and LL presentations and this was followed by stories (21.2%), songs (11.4%), and skits (10.6%) (See Table 1). Some of the poems, stories, songs, and skits are included in Additional file 2.

**Table 1:** Different modes of presentation by the students

Presentation modes	Upper limb muscles *N* = 66	Lower limb muscles *N* = 66	Total *N* = 132
Poem	19 (29%)	30 (45%)	49 (37%)
Story	12 (18%)	16 (24%)	28 (21%)
Song	7 (11%)	8 (12%)	15 (11%)
Skit	10 (15%)	4 (6%)	14 (11%)
Monologue	5 (8%)	3 (5%)	8 (6%)
PowerPoint	3 (5%)	2 (3%)	5 (4%)
Riddle	5 (8%)	0 (0%)	5 (4%)
Video	3 (5%)	0 (0%)	3 (2%)
Game	1 (2%)	0 (0%)	1 (1%)
Drawing	1 (2%)	1 (2%)	2 (2%)

### Experience of students with active and engaging learning strategies

Thirty-eight students (57.5%) completed the anonymous online feedback questionnaire given after the final exam results. Eighty-four percent of the students reported that they benefitted from using the active and engaging learning strategy for their muscle presentations and 92% recommended such sessions for future cohorts. Sixty-three percent agreed that the muscle presentations should include a social message as they were more receptive to such presentations, and this helped in better retention and recollection of facts. When asked about the most preferred method of presentation which helped students to retain and recall the gross anatomical concepts and terminologies, they identified poems (26%), jingles (21%), songs (21%), skits (13%), mimicry (8%), games (5.25%), and posters (2.6%).

Students also found the active and engaging learning strategy to be helpful for the following reasons: encourages creativity, peer to peer interaction and research; active and memorable way to learn; integrated and quick way to gain knowledge; new modes of the presentations by peers made learning easy in a relaxed environment; and live presentations helped in long term memory and retention. The changes students wanted in the future were more time for preparation, competition/quizzes with prizes, and professional recording of presentations for future use.

### Relationships of active and engaging learning strategy with assessment modalities and students’ marks

All raw scores are converted to 100% and the mean scores for all the examinations are shown in Table 2. Comparison of pre-presentation spotter and post-presentation spotter scores of UL revealed that post-presentation scores significantly increased in comparison to pre-presentation (t_65_ = 8.018, *p* < 0.01). This suggests that the intervention strategy used contributed significantly to enhancing the students’ learning. The Pearson correlation demonstrated a significant (*p <* 0.01*)* relationship between different modalities of assessment (Table 3). Further, it could be posited that there is a highly significant positive relationship between:

(i) Pre-presentation spotter (UL) and post-presentation spotter (UL)/mid-term/spotter (LL)/final course marks
(ii) Post-presentation spotter (UL) and mid-term/spotter (LL)/total course marks
(iii) Midterm and spotter (LL)/total course marks
(iv) Spotter (LL) and total course marks
(v) Final examinations and total course marks. However, there was no significant relationship between final examinations scores and any of the modalities of assessment.

**Table 2:** Student performance in examinations (N-66)

Examinations	Weightage	Marks (Mean ± SD)
Pre-presentation spotter (UL)	–	54.5 ± 18
Active and engaging learning presentation (UL)	5%	77.0 ± 16.1
Post-presentation spotter (UL)	5%	67.9 ± 13.6
Mid-Term Exam	20%	66.1 ± 13.4
Active and engaging learning presentation (LL)	5%	72.3 ± 16.4
Spotter (LL)	5%	67.9 ± 13.1
Final Exam	60%	66.6 ± 9.9
Total marks	100%	67.2 ± 8.9

*UL* Upper limb, *LL* Lower limb

**Table 3:** Correlations between different assessment modalities

	Post-presentation spotter (UL)	Mid-term	Spotter (LL)	Final exam	Total marks
Pre-presentation spotter (UL)	0.653^a^	0.516^a^	0.653^a^	0.391^a^	0.550^a^
Post- presentation spotter (UL		0.732^a^	1.000^a^	0.443^a^	0.688^a^
Mid-Term			0.732^a^	0.355^a^	0.719^a^
Spotter (LL)				0.443^a^	0.688^a^
Final Exam					0.884^a^

^a^Correlation is significant at the 0.01 level (2-tailed)

*UL* Upper limb, *LL* Lower limb

### Comparison of performance between student groups

Table 4 shows the comparison of performance between two student Groups A and B. None of the findings were found to be statistically significant, which indicated that students were homogenous and randomly assigned to both the groups.

**Table 4:** Comparison of performance between group A (n_1_ = 33) and Group B (n_2_ = 33)

	Group	Mean ± SD	t-ratio	*p-*value	95% Confidence Interval	
					Lower	Upper
Pre-presentation spotter (UL)	A	57.35 ± 15.18	1.31	0.20	−3.01	14.53
	B	51.60 ± 20.14			−3.03	14.54
Active and engaging learning presentation (UL)	A	77.88 ± 17.28	0.46	0.65	−6.14	9.78
	B	76.06 ± 14.99			−6.14	9.78
Post-presentation spotter (UL)	A	68.40 ± 14.28	0.28	0.78	−5.56	7.37
	B	67.49 ± 11.91			−5.57	7.38
Mid Term	A	67.95 ± 14.19	1.14	0.26	−2.80	10.31
	B	64.19 ± 12.41			−2.80	10.31
Active and engaging learning presentation (LL)	A	71.21 ± 14.74	0.52	0.60	−10.25	6.01
	B	73.33 ± 18.14			−10.26	6.01
Spotter (LL)	A	68.40 ± 14.28	0.28	0.78	−5.56	7.37
	B	67.49 ± 11.91			−5.57	7.38
Final Exam	A	66.79 ± 8.68	0.17	0.86	−4.48	5.33
	B	66.36 ± 11.12			−4.49	5.34
Total	A	67.97 ± 8.48	0.66	0.51	−2.93	5.84
	B	66.52 ± 9.32			−2.93	5.84

*UL* Upper limb, *LL* Lower limb

## Discussion

The present study demonstrated that students found the active and engaging learning strategy beneficial. It helped them to use and apply knowledge in a constructive, active and interesting way, and to exploit their own creativity, curiosity and intelligence. Moreover, students achieved significantly better scores in post-presentation spotters (*p* < 0.01) and the marks of different modalities of assessment also showed a significant positive relationship (*p* < 0.01) possibly due to the adaptation of the active and engaging learning strategy. Many recent studies have used new complimentary technology-based innovative teaching techniques with clinically focused and diversified pedagogical approaches in basic science teaching to fill this gap [2, 3, 5, 17, 29]. The active and engaging learning strategy gives the student overall responsibility for and freedom to create presentations which are easy to understand, remember, and recollect for themselves and their peers [29]. Moreover, students gain confidence as they play the role of the teachers to their peers.

The students chose to use a number of visual, literary, and performing arts-related presentation approaches like poems, stories, songs, skits, monologues riddles, videos, PowerPoint presentations, games, drawings, posters, cartoons, etc. (Table 1, Additional file 2). One of the interesting observations was, very few students chose PowerPoint (3.9%), which is considered to be a widely used presentation mode in medical education [30]. Studies have shown that using such approaches in medical education can improve medical students’ critical, observational, and diagnostic skills [30, 31]. Essential humanitarian instincts and other generic skills (e.g. presentation techniques, communication skills, time management, creativity, etc.) should receive due attention in medical training because they offer many benefits in relation to medical practice later in professional life [32]. Recently, the role of the humanities in training medical students has been garnering recognition, especially the use of ‘literature, art, creative writing, drama, film, music, philosophy, ethical decision-making, anthropology and history’ [33]. Moreover, assessment strategies need to focus on evidence of achievement rather than the ability to regurgitate information, that is, assessment *for*, and not just *of*, learning [34]. Medical schools should put more emphasis on assessment tools that measure not just recall of facts, but the development of the desired competencies needed for future professional life [35].

In our study, more than one-fifth (21.2%) of the students adopted the mode of storytelling in their presentations, which followed the most preferred choice ‘poems’ (37.1%). Students also utilized other modes of presentations as shown in Table 1. According to Kieser et al., spontaneous storytelling approach nurtures reflective learning while students work on their clinical anatomy problems [36]. Poetry was also found to relate well to medicine, where the physician first grasps and then controls the reality of the human predicament [37]. Medical educators can learn about the emotional reactions and concerns of students through their reflective writing in general and poetry in particular [38]. Creative writing offers nurses and doctors a hands-on experience to understand the patient’s hesitancy and degree of difficulty in telling their story and to appreciate the role of feedback, editing, and rewriting in formulating a well-written clinical history [39]. Dolberry (2011) reported the benefits of writing skits and role-playing in a student-guided assignment where instructors transfer more responsibility and independence in choosing to present the information students learnt in the course. Creative writing and role-playing may help students retain course information, expand their knowledge and relate to content in a meaningful way [40].

Many studies at the pre-university level have demonstrated positive outcomes in student perceptions, and, in some cases, class performance, through the use of educational songs and music videos in food service and food safety [41], sciences [42], and natural history [43] curricula. Apart from aiding memorization, songs may potentially improve learning by helping students feel relaxed and welcome in stressful settings, and also by engaging students through multiple modes (verbal vs. nonverbal) and modalities (auditory vs. visual vs. kinesthetic). Game-based learning offers students the opportunity to improve and maintain information literacy skills, particularly information seeking and critical appraisal, in an engaging way [44]. Medical theatre utilized multisensory hands-on learning (e.g. a story, a play script, a poem, a song, use of Play-doh, body painting, a game, a monologue, cartoons, comics, or through role play) and found it to be effective in encouraging active and engaging learning [11].

Change in medical education is a worldwide agenda and many medical schools have witnessed experimentation and experienced challenges [45]. Similarly, an active and integrated approach to the teaching of anatomy has been emphasized in a modern medical curriculum [46]. Many studies have demonstrated the benefits of multimodal teaching modalities by integrating modernized digital technology, dissection, and problem-based learning in anatomy and other fields [46]. However, one of their main drawbacks is that they deliver one-sided information through passive learning, and encourage rote memorization of names and structures; as a result, students fail to develop critical thinking skills [47]. A well-designed active and engaging learning strategy carried out in a suitable and relaxed environment can help to overcome these challenges and limitations.

### Limitations

Our study has a number of limitations. Firstly, the study had a small sample size; therefore, caution needs to be taken in generalizing the data to other settings. Secondly, the effectiveness of the individual modes of presentations was not evaluated. Lastly, the students’ marks may have been influenced by other teaching strategies (lectures, practicals, etc.) or by students’ previous knowledge. Further studies should be conducted utilizing multiple campuses of The UWI to explore the effectiveness of the various modalities of the active and engaging learning strategy in anatomy, and in other disciplines as well.

### Conclusion

Our study highlighted that the active and engaging learning strategy can be used as an effective learning tool to teach anatomy. This strategy helped students to take an active role in learning and utilizing their own creativity, curiosity, and intelligence. The core curriculum for musculoskeletal anatomy needs to be identified for undergraduate and post-graduate curricula, and innovative teaching techniques should be adopted for effective and interactive learning. As research into the usefulness and evaluation of active and engaging learning strategy is still in its infancy in anatomy teaching, further research to assess the effectiveness of each modality of the active and engaging learning strategy would provide a better approach to teaching anatomy. Use of randomized controlled trials to assess the effectiveness of various learning strategies could open a new door to medical education.

## Additional files

Additional file 1: Scoring Rubric for Presentations. (DOCX 15 kb)

Additional file 2: Selected Presentations by the students. (DOC 393 kb)

## Abbreviations

CAL: Computer-assisted learning
CBL: case-based learning
FMS: Faculty of Medical Sciences
LL: Lower limbs
MBBS: Bachelor of Medicine and Bachelor of Surgery
MCQ: Multiple choice questions
SAQs: Short answer questions
TBL: Team-based learning
The UWI: The University of the West Indies
UL: Upper Limb
USMLE: United States Medical Licensing Examination

## Authors' information

KEERTI SINGH, MBBS, MS (Anatomy) is a Lecturer of Anatomy in the Faculty of Medical Sciences, The UWI Cave Hill Campus, Barbados. She teaches clinical anatomy, embryology and histology to the medical students in Phase 1. Her research interest is in medical education, gross anatomy, clinical anatomy, and male infertility.

AMBADASU BHARATHA, MSc, Ph.D. (Pharmacology) is a Lecturer of Pharmacology in the Faculty of Medical Sciences, The UWI, Cave Hill Campus, Barbados. He teaches pharmacology to the MBBS students in Phase 1. His research areas of interest are experimental pharmacology, obesity, diabetes, and cardiovascular pharmacology.

BIDYADHAR SA BA (Hons), MA, MPhil, Ph.D. (Education) is the Head of the Centre for Medical Sciences Education and Deputy Dean, Quality Assurance and Accreditation, Faculty of Medical Sciences, The UWI, St Augustine Campus, Trinidad & Tobago. His main research interests include assessment in medical education, emotional intelligence, and empathy among health professional students and practitioners.

OSWALD PETER ADAMS BSc, MBBS, MSc, DM (Family Medicine) is the Dean of the Faculty of Medical Sciences, The UWI, Cave Hill Campus, Barbados. He is a Family Physician. His main research areas are chronic non-communicable diseases and sexually transmitted infections.

MD. ANWARUL AZIM MAJUMDER MBBS, MHPEd, PGDipHEcons, FHEA, Ph.D. (Medical Education) is the Director of Medical Education of the Faculty of Medical Sciences, The UWI Cave Hill Campus, Barbados. His main research interests are in medical/health profession education, health systems, and public health, and he has extensive experience in curriculum development, HRH management, and implementing faculty development programs in developed and developing countries.
