# Supplementary material for: Teaching anatomy using an active and engaging learning strategy
Source: BMC Med Educ. 2019 May 16;19:149. doi: 10.1186/s12909-019-1590-2 (PMC6524257; doi:10.1186/s12909-019-1590-2)
Supplement: Supplementary file 1 — Scoring Rubric for Presentations. (DOCX 15 kb) [file 12909_2019_1590_MOESM1_ESM.docx]

Additional file 1

**Scoring rubric of the presentations**

Mark distribution:

- Creativity: 2 marks
- Relevance: 2 marks
- Accuracy: 2 marks
- Completeness: 2 marks
- Confidence: 2 marks
- Total: 10 marks

| **Scoring Criteria** |  | **Total Marks** | **Score** |
| --- | --- | --- | --- |
| Creativity | - Information is presented in a logical sequence. - Visual aids are well prepared, informative, effective, and not distracting. | 2 |  |
| Relevance | - The type of presentation is appropriate for the topic and audience. - Material included is relevant to the overall message/purpose. | 2 |  |
| Accuracy | - Technical terms are well-defined in language appropriate for the target audience. - Presentation contains accurate information. | 2 |  |
| Completeness | - Introduction is attention-getting, lays out the problem well, and establishes a framework for the rest of the presentation. - Appropriate amount of material is prepared, and points made reflect well their relative importance. - There is an obvious conclusion summarizing the presentation. - Length of presentation is within the assigned time limits. | 2 |  |
| Confidence | - Speaker maintains good eye contact with the audience and is appropriately animated (e.g., gestures, moving around, etc.). - Speaker uses a clear, audible voice. Good language skills and pronunciation are used. - Good language skills and pronunciation are used. - Delivery is poised, controlled, and smooth. Information was well communicated. | 2 |  |
| **Total** | | **10** |  |
