# Supplementary material for: Teaching anatomy using an active and engaging learning strategy
Source: BMC Med Educ. 2019 May 16;19:149. doi: 10.1186/s12909-019-1590-2 (PMC6524257; doi:10.1186/s12909-019-1590-2)

**Additional file 2**

**Selected presentations by the students**

1. **Story:**

*Title: Gluteus Maximus’ Dream Job*

Maximus is one of the muscles of the superficial gluteal muscle group. Maximus is larger than all the other muscles, so he takes up almost all the space on the posterior aspect of the hipbone except the superolateral aspect where Gluteus Medius sits. He grew up on the crest and posterior surface of the ilium just posterior to the posterior gluteal line. When he was four, he then moved to the dorsal surfaces of the sacrum and the coccyx and he played at the Sacrotuberous ligament park. A few months ago, the company Maximus was working for split up to insert into different points and he got laid off. His superior and superficial fibres inserted into the iliotibial tract and indirectly to the linea aspera on the femur. Meanwhile, his deep and inferior fibres inserted into the gluteal tuberosity of the femur.

One day Maximus got a call from the L5, S1 and S2 roots of the Inferior Gluteal Nerve Inc. They told him that he had gotten his dream job and he got very excited. So excited that he began to contract and powerfully extend the thigh at the hip joint. However, Max had to take a test to prove his proficiency in laterally rotating the thigh at the hip joint and stabilizing the tibia while standing.

Fortunately, he passed the test and moved on to his next step in securing the job, which was a check-up at the doctor. However, on the date the appointment was scheduled for Max came down with a very painful case of trochanteric bursitis and had to postpone it. A few days later, he was able to get his check-up done and it was discovered that he was missing an important intramuscular injection. He had to be given it in his upper lateral quadrant to avoid damage to the sciatic nerve. Finally, Maximus was able to get the job. Unfortunately, he soon realized that it was more stress than his ischial bursa could handle and came down with a serious case of chronic ischial bursitis with calcification of his bursa. He had to take a few sick days and when he got better the first thing he did was to go to his favorite restaurant the Inferior Gluteal Artery. He ordered a superior gluteal artery blood supply and so the next day he was back to working at the hip joint again.

1. **Song:**

*Title: Obturator Internus Song*

I’m a lower limb muscle can you guess who I am?

I’m deep and posterior, let’s see if you can.

The obturator foramen and membrane is where I was born,

The medial surface of the greater trochanter is what I insert on.

My main action is to laterally rotate the thigh,

The medial circumflex femoral artery is where I get blood supply.

I’m innervated by nerve roots L5, S1 and even S2,

I’m sure by now you’ve already guessed who.

Injury to this muscle results in excruciating groin pain,

It’s common in football athletes and known as a muscle sprain.

I forgot to mention 4 other muscles with whom i have a great friendship

Collectively we are known as lateral rotators of the hip.

Superior and inferior gemellus, obturator externus and piriformis,

That’s right you’ve guessed it, my name is Obturator internus!

1. **Skit:**

*Title: A dialogue between Obturator Externus and Adductor Magnus*

*Adductor Magnus flexing in front of a mirror*: I am the greatest! I am the largest and most powerful muscle in the posterior adductor group... How would they ever survive without my expertise! I am such a dynamic and versatile guy, so much so that I am composed by two parts: my adductor half which originates from the inferior ramus of pubis and ramus of ischium. And then there is my hamstrings half, which originates from the ischial tuberosity, for the ladies that prefer to get in touch with my more feminine side.

*Obturator externus looks on as Adductor magnus adores himself:*

Oh, how I wish I was as amazing and had as much confidence as Adductor Magnus. I am such a small and insignificant part of the thigh and no one cares to acknowledge my actions as a muscle. If I were to disappear now, no one would even realize I was gone.

*Adductor Magnus overhears the little boy’s thoughts of himself and immediately approaches him with concern:*

I never knew you felt this way son.

*Obturator externus:* Well it’s true. The other guys always make fun of my size and tell me that there is hardly any need for me.

*Adductor Magnus:* I would have you know that I have always had admiration for all the great things you of the hip joint and the other short muscles do. You have never allowed your size to dictate your work and honestly, even I couldn’t measure up to that.

*Obturator externus:* Oh really Mr. Magnus?! This has to be the first time anyone has ever said such nice things to me.

*Adductor Magnus:* Come here son, (encourages him to step forward to the mirror):

Which guy here has an entire nerve named after him?

*Obturator externus:* Hahahaha, that would be me! (he replies enthusiastically)

*Adductor Magnus:* Yesssss, it sure is! Bet no one told you it supplies the adductor part of me as well!

*Obturator externus:* It does?! I would have never figured. I always thought it was the tibial part of the Sciatic nerve....

*Adductor Magnus:* Well, the tibial nerve only supplies my Hamstring’s portion. And now for my next question.... Who here has their sites of origin named after them?

*Obturator Externus:* Oh wow, that would be me again! (says the young boy as he giggles excitedly) At the margins of the obturator foramen and obturator membrane of the hip bone!

*Adductor Magnus:* Exactly! So, there you have it... You are very much worthy and strong as a lateral rotator of the thigh son (patting the boy on the head).

*Obturator Externus:* I also help to stabilize the head of the femur in the acetabulum! (Obturator says assertively).

*Adductor Magnus:* Hahahaha, oh yes you are indeed, how could I forget. Well I see you do feel a lot better now.

*Obturator Externus:* Definitely Sir! This meeting surely meant a lot to me and I greatly appreciate it.

*Adductor Magnus:* Well that’s wonderful to hear. We’ll chat sometime again soon. It should be easy since you right over there posterior to the pectineus and the superior ends of the adductor muscles. And we’re both located in the medial compartment, so I’ll definitely see you around Skipper!

*Obturator Externus:* Sure thing! So long Mr. Magnus, till next time.

1. **Monologue:**

We had to flex on them

So, everyone keeps saying we need these

Opposable thumb this opposable thumb that

But the fact it without them next 4 digits we wouldn’t be able to pick up keys for less for holding them

You see people does try and down play our importance but we have to come and show them our significance

We have to flex

Who me, well my anatomical name is flexor digitorum superficialis

But you can call me flex for short

With meh brethren Dus or flexor digitorum profundas

That’s Dus with a D not with a t

He does pronate but that have nothing to do with me

You can’t see him because he deep than me

Ladies I know you like your deep muscles so you can check him after

See what we do we does flex

With four digits we regret it could have been five but the longest one we Doh talk about he

Where we come from you see we have two origins cause I have two heads

Ladies two heads

I have two heads

I started from the epicondyle not the lateral but the medial

We doh hadda talk about the lateral Doh extensors will know what I am talking about

I make my way down the margin of the CP

Coronoid process that is what we call it on my end

Then we end up on the anterior of the superior boarder of the radius

Sorry about the technical terms you might here a few technical terms in between

I make my way down the CP and meet my friend Dus

Ladies don’t forget about Dus

He originate from the medial anterior surface of the interosseous membrane

That fibrous sheath between the radius and the ulnar

Attached to their margins like Caribbean people and their culture

Where are we now?

They say we started from the bottom now we here

Clearly that’s not the case cause we started from the proximal and ended on the base

Dus inserts himself on the base of the distal phalanges of the middle four digits

And I insert myself on the shaft of the same four digits

But we still repping the four to the world

Unlike the thumb we can actually please a girl

Everyone need some support

We are supplied by the median artery

Doh tell Dus I say this but he kinda needy

He have two different blood supplies and two different nerve supplies

The ulnar and the anterior interosseous arteries are one thing

But u see when you have the anterior interosseous nerve on the 2^th^ and the 3^th^ digit

And the ulnar nerve on the 4^th^ and 5^th^ digit bruh!

That’s doing too much

What we does do

You should know this by now

We flex.

1. **Poem:**

*Title: Anconeus*

You’re small triangular elbow muscle not a boneus

You originate from the lateral epicondyle of the humerus

If I told you all the things I love about you it would be limitless

You insert into the olecranon process of the ulnar bone

Innervated by the radial nerve c7 c8 t 1 my mind was blown

However, you only have one action and I don’t want to cause you harm

But your only purpose is extension of the forearm

Sorry anconeus but I met someone new

His name is extensor carpi ulnaris and he comes from the lateral epicondyle too

Unlike you he inserts into the base of the fifth metacarpal

Hope this does not come of shock

Don’t want to make you stress out

He’s innervated by the radial nerve c7 c8 t1

Don’t want to break your heart but he’s a keeper

You anconeus are supplied by the interosseous anterior artery

While he is supplied by the ulnar

I hope you get my point

You only extend my elbow

He extends and adducts my hand at my wrist joint

So good bye anconeus you small triangular muscle of my elbow

I found another muscle on the other side of my forearm

So here I go

1. **PowerPoint**

*Title: WHO AM I?*

**
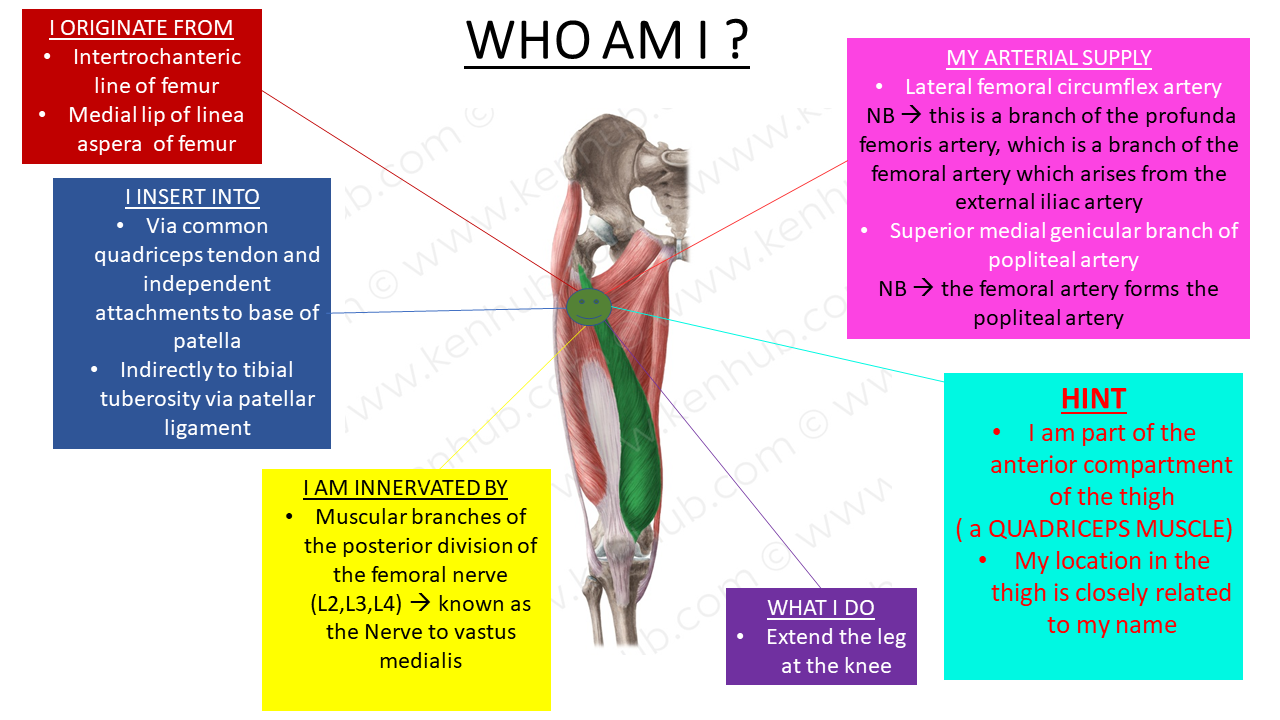
**

[Image source attribution: © Kenhub ([https://www.kenhub.com](https://www.kenhub.com/)); Illustrator: L. Znotina]

**7) Game:**

*Title: Hangman*


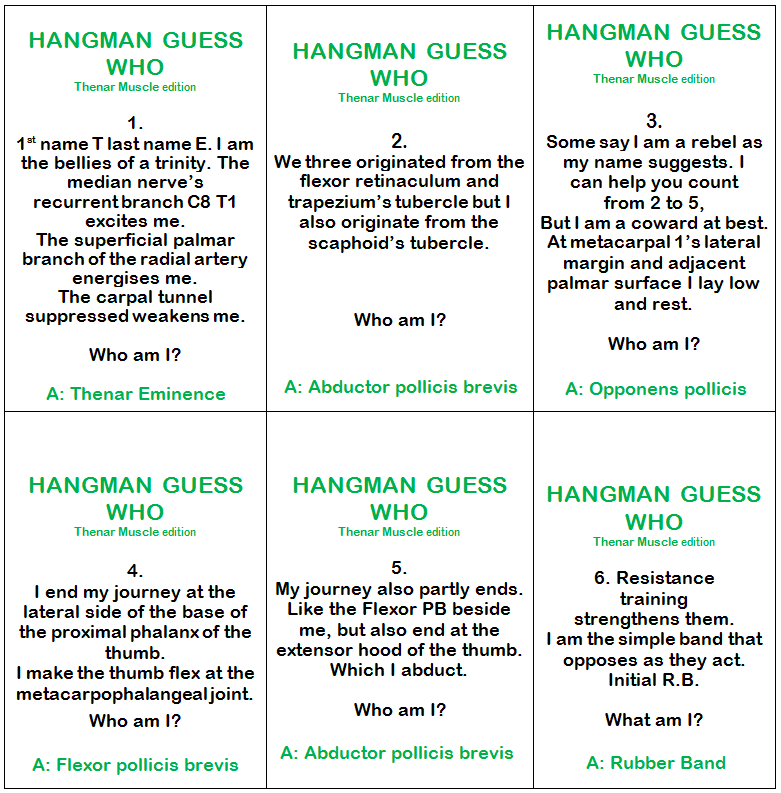

Supplement: Supplementary file 2 — Selected Presentations by the students. (DOC 393 kb) [file 12909_2019_1590_MOESM2_ESM.doc]
